# Supplementary material for: Chitosan Film Sensor for Ammonia Detection in Microdiffusion Analytical Devices
Source: Polymers (Basel). 2023 Oct 27;15(21):4238. doi: 10.3390/polym15214238 (PMC10650627; doi:10.3390/polym15214238)
Supplement: Supplementary file 1 [file polymers-15-04238-s001.zip › polymers-2680398-supplementary.pdf]

SEM

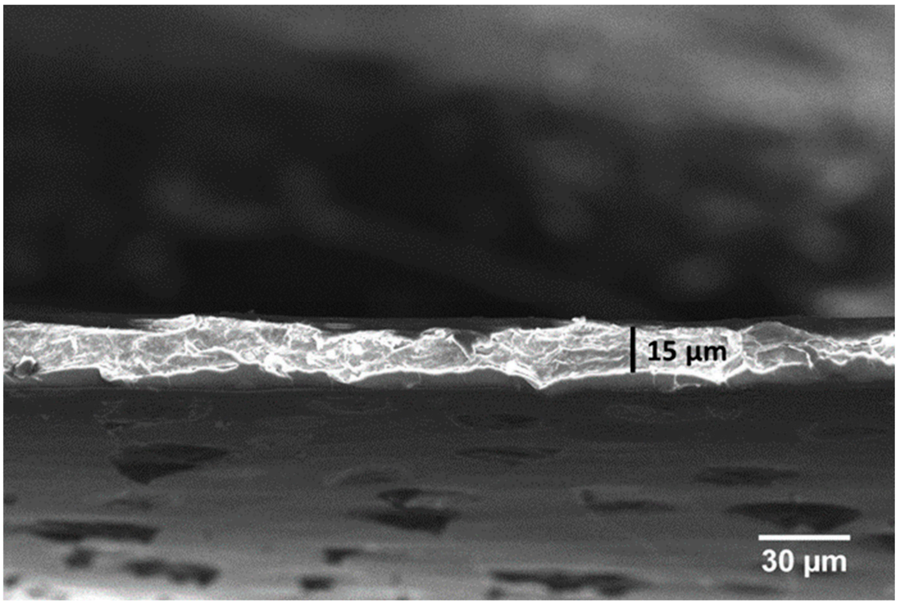

Figure S1. SEM image of a cross section of CSF. Measured thickness 15 μm.

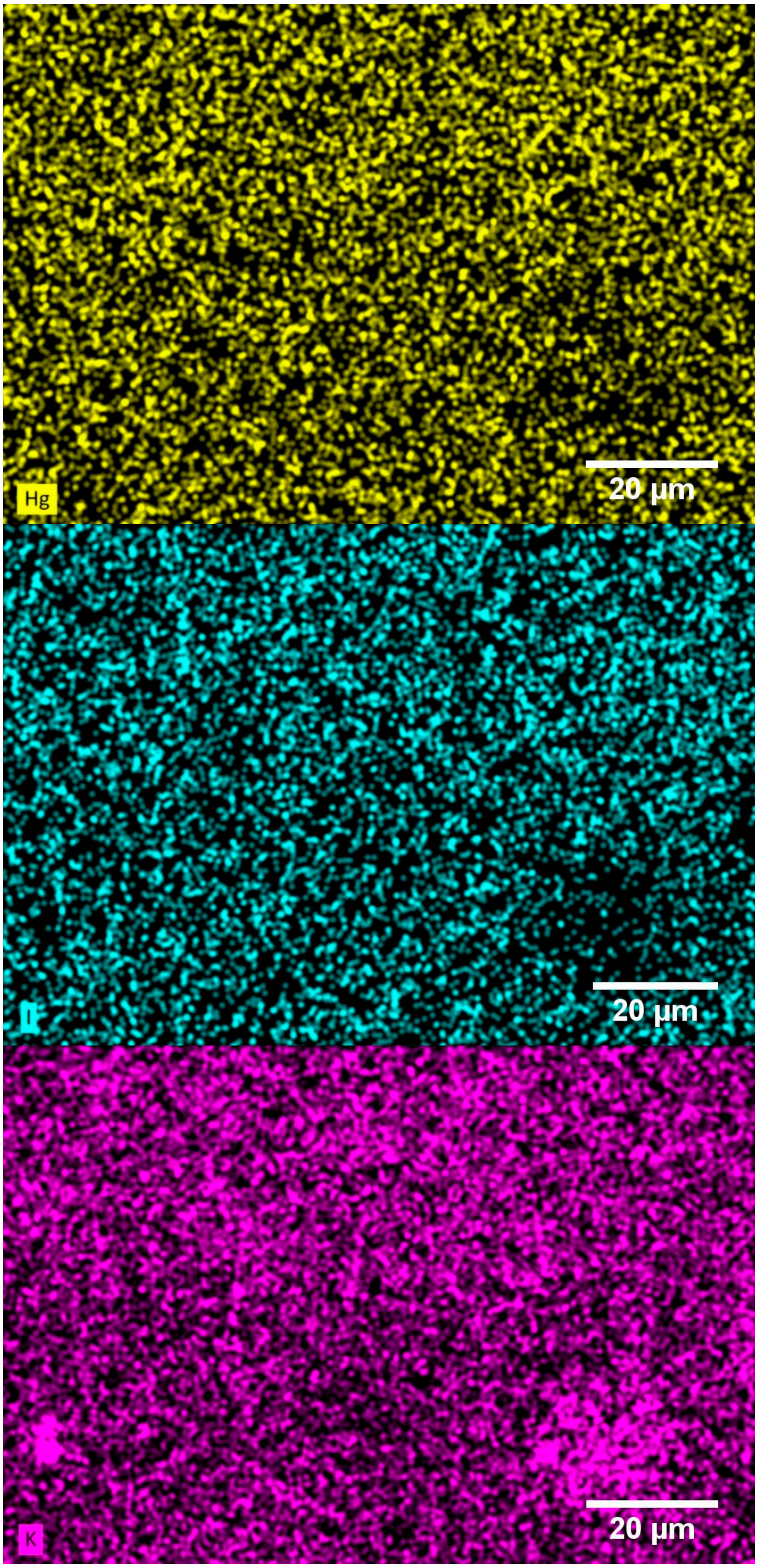

Figure S2. EDX analysis of a CFS for single elements.
